# Genetic Virulence Profile of Enteroaggregative *Escherichia coli* Strains Isolated from Danish Children with Either Acute or Persistent Diarrhea

**DOI:** 10.3389/fcimb.2017.00230

**Published:** 2017-05-30

**Authors:** Betina Hebbelstrup Jensen, Anja Poulsen, Stig Hebbelstrup Rye Rasmussen, Carsten Struve, Jørgen H. Engberg, Alice Friis-Møller, Nadia Boisen, Rie Jønsson, Randi F. Petersen, Andreas M. Petersen, Karen A. Krogfelt

**Affiliations:** ^1^Department of Bacteria, Parasites and Fungi, Statens Serum InstitutCopenhagen, Denmark; ^2^Department of Gastroenterology, Copenhagen University Hospital HvidovreCopenhagen, Denmark; ^3^Department of Political Sciences and Public Management, University of Southern DenmarkOdense, Denmark; ^4^Department of Clinical Microbiology, Slagelse HospitalSlagelse, Denmark; ^5^Department of Clinical Microbiology, Copenhagen University Hospital HvidovreCopenhagen, Denmark

**Keywords:** enteroaggregative *Escherichia coli*, EAEC, childhood diarrhea, virulence factors, risk factors

## Abstract

Enteroaggregative *Escherichia coli* (EAEC) is frequently found in diarrheal stools worldwide. It has been associated with persistent diarrhea, weight loss, and failure to thrive in children living in developing countries. A number of important EAEC virulence genes are identified; however, their roles in acute and persistent diarrhea have not been previously investigated. The aim of this study was to identify specific EAEC virulence genes associated with duration and type of diarrhea in Danish children. We aimed to improve the current diagnostics of EAEC and enable targeting of strains with an expected severe disease course. Questionnaires answered by parents provided information regarding duration of diarrhea and presence of blood or mucus. A total of 295 EAEC strains were collected from children with acute (≤7 days) and persistent diarrhea (≥14 days) and were compared by using multiplex PCR targeting the genes *sat, sepA, pic, sigA, pet, astA, aatA, aggR, aaiC, aap, agg3/4C, ORF3, aafA, aggA, agg3A, agg4A*, and *agg5A*. Furthermore, the distribution of EAEC genes in strains collected from cases of bloody, mucoid, and watery diarrhea was investigated. The classification and regression tree analysis (CART) was applied to investigate the relationship between EAEC virulence genes and diarrheal duration and type. Persistent diarrhea was associated with strains lacking the *pic* gene (*p* = 0.002) and with the combination of the genes *pic, sat*, and absence of the *aggA* gene (*p* = 0.05). Prolonged diarrhea was associated with the combination of the genes *aatA* and *astA* (*p* = 0.03). Non-mucoid diarrhea was associated with strains lacking the *aatA* gene (*p* = 0.004). Acute diarrhea was associated with the genes *aggR, aap*, and *aggA* by individual odds ratios. Resistance toward gentamicin and ciprofloxacin was observed in 7.5 and 3% of strains, respectively. Multi-drug resistance was observed in 38% of strains. Genetic host factors have been associated with an increased risk of EAEC-associated disease. Therefore, we investigated a panel of risk factors in two groups of children—EAEC-positive and EAEC-negative—to identify additional factors predisposing to disease. The duration of breastfeeding was positively correlated with the likelihood of belonging to the EAEC-negative group of children.

## Introduction

Enteroaggregative *Escherichia coli* (EAEC) is an established pathotype within the group of diarrheagenic *E. coli* (DEC), which also includes the enteropathogenic *E. coli* (EPEC), enterotoxigenic *E. coli* (ETEC), enteroinvasive *E. coli* (EIEC), and verocytotoxin-producing *E. coli* (VTEC). EAEC is associated with diarrhea, failure to thrive, weight loss, and stunted growth in children living in developing countries (Steiner et al., 1998; Albert et al., 1999; Lima et al., 2000; Medina et al., 2010; Hebbelstrup Jensen et al., 2014). EAEC-positive children were seen to have increased levels of fecal lactoferrin and Il-1β, regardless of the presence of gastrointestinal symptoms in a Brazilian study (Steiner et al., 1998). This indicates a considerable inflammation potential of EAEC and severe illness, which may also be present in children in industrialized countries. EAEC has been associated with childhood diarrhea in Germany, England and America (Huppertz et al., 1997; Jenkins et al., 2006; Vernacchio et al., 2006). Furthermore, several outbreaks of EAEC have been reported in children in Serbia, Japan and Korea (Cobeljić et al., 1996; Harada et al., 2007; Shin et al., 2015). Persistent diarrhea was described in EAEC-infected children (Bhan et al., 1989), which may result in considerable loss of electrolytes and impaired absorption of micronutrients. Several genetic host factors have been associated with an increased susceptibility toward EAEC infection including single nucleotide polymorphisms (SNPs) in the interleukin-8 promoter region (Jiang et al., 2003) and in the CD14 gene (Mohamed et al., 2011). However, only few general risk factors associated with EAEC infection have been investigated (Hebbelstrup Jensen et al., 2014). Susceptibility testing of EAEC strains has revealed a considerable resistance toward antibiotics, which includes resistance toward ciprofloxacin (Aslani et al., 2011), multi-drug resistance (Khoshvaght et al., 2014), and extended-spectrum beta-lactamases (Guiral et al., 2011), which is a cause for concern. The pathogenic potential of EAEC in children in industrialized countries warrants further research and the role of EAEC virulence factors in acute and persistent diarrhea needs clarification.

The gold standard for the identification of EAEC is by the HEp-2 cell assay. This test is performed in reference laboratories only; it requires cell culture facilities and is time-consuming (Hebbelstrup Jensen et al., 2014). This method is depend on recognition of the so-called “stacked-brick” appearance by special trained personnel and it has been observed to be subject to inter-observer variability (Hebbelstrup Jensen et al., 2014). In addition, this phenotypical assay does not distinguish between pathogenic and non-pathogenic strains. Molecular techniques have been developed to detect pathogenic EAEC strains, and various gene targets have been used. It was originally suggested that EAEC could be detected by the master regulator *aggR* (Morin et al., 2013), which led to a general classification of EAEC based on the presence of the *aggR* gene into typical and atypical strains (Morin et al., 2013), but not all diarrheagenic EAEC strains possessed the *aggR* gene (Jenkins et al., 2007). Conventionally, the genes *aggR, aatA*, and *aap* are used as initial detection for the EAEC virulence plasmid encoding the master regulator, a secretion system and the dispersin protein, respectively (Vial et al., 1988). In addition, the chromosomal gene *aaiC* is frequently used for diagnosis of EAEC. The *aaiC* gene is encoded on a genomic island encoding a type VI secretion system (Dudley et al., 2006). The *ORF3* gene encodes a cryptic protein and it was the most frequently detected EAEC virulence gene in children in Mali (Boisen et al., 2012). In spite of these research findings, no consensus has been reached on which EAEC genes are unambiguously pathogenic.

Colonization of the gut is accomplished by adhesion facilitated by the EAEC aggregative adhesion fimbriae (AAFs) with five variants AAF/I—AAF/V (Czeczulin et al., 1997; Shamir et al., 2010; Jønsson et al., 2015; Nezarieh et al., 2015). An important pathogenic trait of EAEC is the formation of biofilm (Hicks et al., 1996), which is in general associated with persistent infection (Costerton et al., 1999). Biofilm formation has been associated with the genes *aatA, aggR, pic, sepA*, and *sigA* (Mohamed et al., 2007; Mendez-Arancibia et al., 2008; Nezarieh et al., 2015) and the AAFs (Czeczulin et al., 1997; Shamir et al., 2010; Jønsson et al., 2015; Nezarieh et al., 2015). The genes *sat* and the *pet* are toxins that causes considerable damage to the intestinal epithelium (Eslava et al., 1998; Taddei et al., 2005). In a Brazilian study investigating childhood diarrhea, the toxin gene *astA* was associated with acute diarrhea, and the CVD432 probe (corresponding to the *aatA* gene) was associated with persistent diarrhea (Pereira et al., 2007).

The role of EAEC in childhood diarrhea in Denmark has only been investigated sparsely (Olesen et al., 2005; Hebbelstrup Jensen et al., 2016b). To date a number of EAEC virulence genes have been identified, inducing adhesion, cytotoxicity, mucosal inflammation, and immune evasion (Hebbelstrup Jensen et al., 2014). The aim of this study was to investigate the clinical manifestations and risk factors for EAEC infection in Danish children with diarrhea. Specific EAEC virulence genes (Table 1) were investigated for an association with diarrheal type and duration. Improvement of the current diagnostics of EAEC could be achieved by targeting EAEC genes with an expected protracted course of disease. Furthermore, identification of specific EAEC genes associated with diarrheal type would contribute to the general understanding of the pathophysiological mechanisms of EAEC.

**Table 1:** Description of the EAEC genes detected by the multiplex PCR and their function.

	**Number of genes detected (%)**	**Description**	**Function**
*aap*	67 (77)	Dispersin	Anti-aggregation
*aggR*	65 (75)	Transcription activator	Major regulator
*aggA*	14 (16)	Fimbrial subunit for AAF/I	Adhesion
*aafA*	6 (7)	Fimbrial subunit for AAF/II	Adhesion
*agg3A*	6 (7)	Fimbrial subunit for AAF/III	Adhesion
*agg4A*	10 (12)	Fimbrial subunit for AAF/IV	Adhesion
*agg5A*	17 (20)	Fimbrial subunit for AAF/V	Adhesion
*agg3/4C*	30 (35)	Usher	Adhesion
*aatA*	81 (93)	ABC transporter	Transporter of dispersin
*astA*	27 (31)	Aggregative heat-stabile toxin	Toxin
*aaiC*	58 (67)	Type VI secretion system	Secreted protein
ORF3	65 (75)	Cryptic protein	Unknown
*pet*	9 (10)	Plasmid encoded toxin	Toxin
*pic*	45 (52)	Serine protease precursor	Toxin
*sat*	25 (29)	Secreted auto-transporter protein	Toxin
*sepA*	21 (24)	Serine protease auto-transporter toxin	Toxin
*sigA*	3 (3)	Protease-like homolog	Toxin

## Materials and methods

### Study population

In the period between January 2011 and October 2013, we conducted a multi-center study to identify children with diarrhea and EAEC detected in stool samples. The participating diagnostic units in the study were the Department of Bacteria, Parasites and Fungi, Statens Serum Institut, and the Departments of Clinical Microbiology at Slagelse Hospital and Copenhagen University Hvidovre Hospital in Denmark. At Copenhagen University Hvidovre Hospital, only patients suffering from travelers' diarrhea were investigated for EAEC. The stool samples originated from children with diarrhea, who had been consulting their general practitioner or who had been hospitalized. Children with EAEC positive stool samples and no co-infections were eligible for inclusion in the study.

### The group of EAEC-positive children

**Figure 1:**
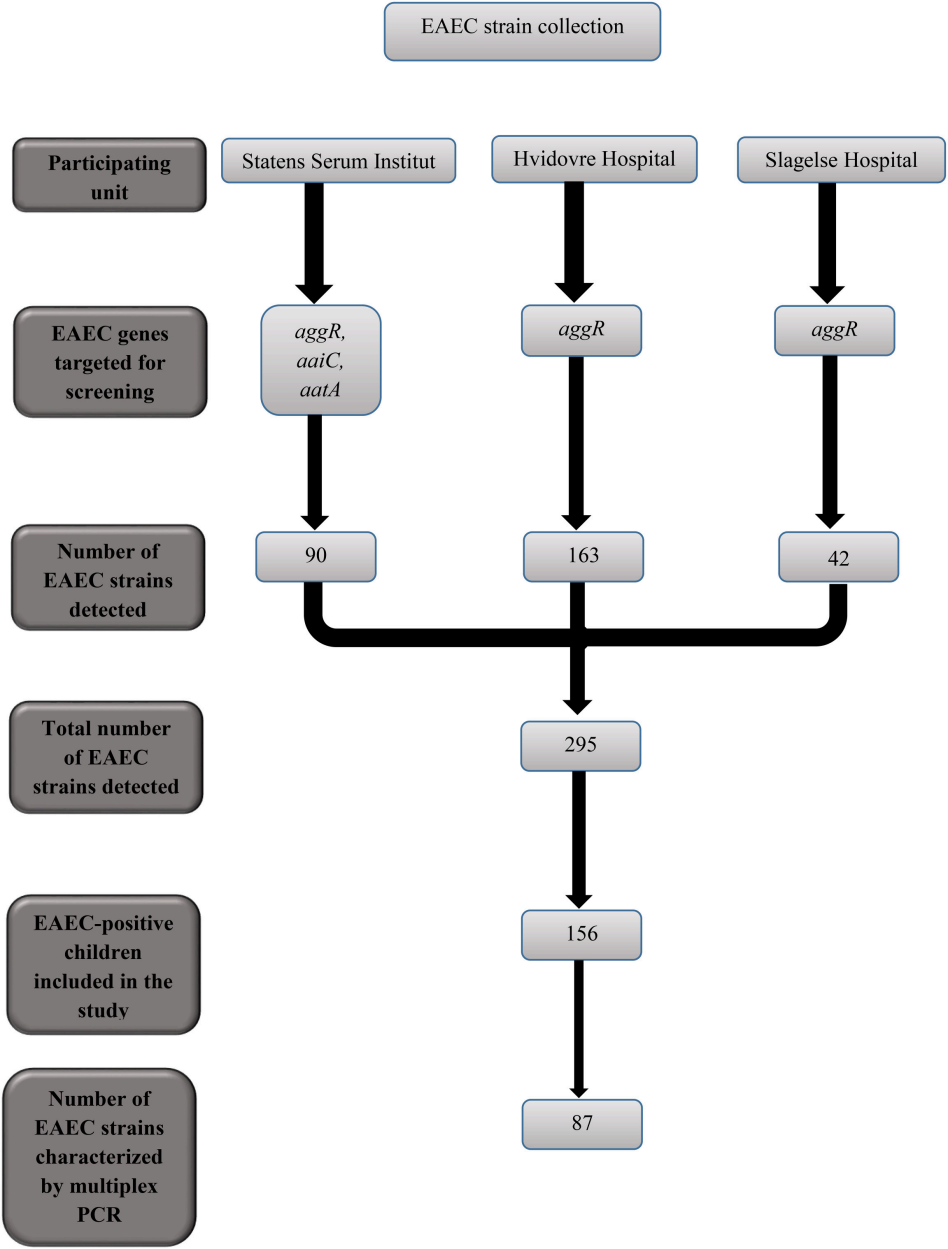
Flow-chart showing the EAEC strains collected from children with diarrhea at the three units participating in the study.

In the period between January 2011 and October 2013, 295 children tested positive for EAEC at the three participating Departments of Clinical Microbiology (Figure 1). From the 295 children, 89 (30%) were excluded due to co-infection with one or several additional enteric pathogens. Among co-infections, the most frequently detected were the attaching-and-effacing *E. coli* (AEEC; *n* = 24), rotavirus (*n* = 12), ETEC (*n* = 12), and *C. difficile* (*n* = 10). From the remaining 206 EAEC-positive children, the parents of 50 children had no phone number listed, could not be contacted, did not speak Danish, or did not live in Denmark and were excluded. We included 156 children positive for EAEC only in the EAEC-positive group.

### The EAEC-negative group of children

The EAEC-negative group consisted of 155 healthy children aged 0–6 years, who were included in a cohort study, we had conducted in the period between 2009 and 2013 (Hebbelstrup Jensen et al., 2016a,b; Jokelainen et al., 2017). In that study, we had investigated the incidence and pathological significance of EAEC in Danish children in municipal daycare in the Copenhagen area. Each child had been observed for a 1 year period with registration of gastrointestinal symptoms and submission of stool samples. We included children in the EAEC-negative group from the cohort at their first point of observation. Six EAEC-positive children were excluded from the EAEC-negative group, where two children had reports of diarrhea or vomiting. In the remaining 155 EAEC-negative children, 34 had reports of diarrhea. From the 34 children with diarrhea the following pathogens were detected: norovirus (*n* = 7), sapovirus (*n* = 4), rotavirus (*n* = 3), adenovirus and AEEC (*n* = 2), sapovirus and norovirus (*n* = 1). AEEC, EPEC, VTEC, and *C. difficile* were each detected in one child (Table 2). No pathogens were detected in 13 children with diarrhea in the EAEC-negative group. The characteristics of the EAEC-positive and the EAEC-negative groups are presented in Table 3.

**Table 2:** Diarrhea was reported from 34 children in the EAEC-negative group and 21 children were diagnosed with an enteric pathogen.

**Microorganism**	**%**	**(No. #)**
**ENTERIC MICROORGANISMS IN THE EAEC-NEGATIVE GROUP WITH**		
**DIARRHEA (*****N*** = **34)**		
Norovirus	21	(7)
Sapovirus	4	(4)
Rotavirus	9	(3)
Adenovirus + AEEC	6	(2)
Sapovirus + Norovirus	3	(1)
AEEC	3	(1)
EPEC	3	(1)
*C. difficile*	3	(1)
VTEC	3	(1)

*No microorganism was detected in 13 children with diarrhea*.

**Table 3:** Characteristics of the EAEC-positive group of children and the EAEC-negative group of children.

	**EAEC-positive group *n* = (156)**	**EAEC-negative group *n* = (155)**	***P*-value with confidence interval**
**AGE IN YEARS**			
0–2	102 (65%)	78 (50%)	*P* = 0.5404^a^
3–5	38 (24%)	73 (47%)	[−0.6; 0.3]
6+	15 (10%)	4 (3%)	
Median age	2	2	
**GENDER**			
Boys	92 (59%)	87 (56%)	*P* = 0.4326^b^
			[−0.1; 0.2]
**Mean birth weight**	3516 g	3414 g	*P* = 0.1692
			[−43.4; 246.3]^a^
**HISTORY OF INFANTILE COLIC**			
Yes	26 (17%)	18 (12%)	*P* = 0.2008^b^
No	130 (83%)	137 (88%)	[−0.1; 0.1]
**BREASTFEED CURRENTLY**			
Yes	9 (6%)	3 (2%)	*P* = 0.0792^b^
No	138 (94%)	146 (94%)	[−0.0; 0.1]
**PREVIOUSLY BREASTFED**			
Yes	131 (84%)	142 (92%)	
No	16 (10%)	7 (4%)	
Not answered	9 (6%)	6 (4%)	
**BREASTFEEDING DURATION**			
>0–5 months	35 (22%)	23 (15%)	*P* = 0.0017^a^
6–9 months	49 (31%)	43 (28%)	[−2.7; 0.6]
10–12+ months	42 (27%)	69 (45%)	
Not answered	30 (19%)	20 (13%)	
**DOMESTIC ANIMAL**			
Yes	37 (24%)	33 (21%)	*P* = 0.9368^b^
No	119 (76%)	118 (75%)	[−0.1; 0.1]
Unanswered	0	7 (4%)	
**DIARRHEA**^c^			
Yes	156 (100%)	34 (22%)	
No	0 (0%)	115 (74%)	
Not answered	0 (0%)	6 (4%)	
**HOSPITALIZED**			
Yes	19 (12%)	0	
**CONTACT WITH SICK ANIMALS**^c^			
Yes	7 (5%)	9 (6%)	*P* = 0.5351^b^
No	118 (76%)	115 (74%)	[−0.2; 0.1]
Unsure	31 (20%)	28 (18%)	
Unanswered	0 (0%)	3 (2%)	
**FOREIGN TRAVEL**^c^			
Yes	79 (51%)	26 (17%)	*P* = 0.0000^b^
No	76 (49%)	128 (83%)	[0.2; 0.4]
Not answered	0 (0%)	1 (1%)	
**USE OF ANTIBIOTICS**^c^			
Yes	26 (17%)	28 (18%)	*P* = 0.4798^b^
Penicillin	14 (9%)	24 (16%)	[−0.1; 0.1]
Macrolides	2 (1%)	0 (0%)	
Other	1 (1%)	0 (0%)	
Unknown	4 (3%)	3 (2%)	
No	128 (82%)	126 (81%)	
Not answered	1 (1%)	1 (1%)	

a *Independent sample t-test comparing the duration of breastfeeding between the study groups*.

b *Difference in proportions test*.

c *Within a period of 2 months prior to sampling*.

*N/A, not applicable*.

### Microbiological analysis

Stool samples were analyzed for the enteropathogens as a part of the routine diagnostics at the participating Departments of Clinical Microbiology. Culturing of the stool samples was performed by using the SSI selective enteric medium (Blom et al., 1999) for detection of *Salmonella* spp., *Yersinia* spp., *Shigella* spp., *Vibrio* spp. *Aeromonas* spp., and *E. coli* spp. For identification of *Campylobacter* spp. and *C. difficile* the modified charcoal cefoperazone deoxychocolate agar medium was used (Hutchinson and Bolton, 1984). The microbiological analysis included PCR for detection of ETEC, VTEC, EPEC, EIEC, AEEC, *Aeromonas* spp., and *C. difficile* (Persson et al., 2007, 2008, 2015). *E. coli* colonies with different morphology were handpicked and were sub-cultured on MacConkey agar. To diagnose EAEC, the genes *aatA* (dispersin transporter protein), *aggR* (transcription activator), and *aaiC* (secreted protein) were targeted by PCR at the Department of Bacteria, Parasites, and Fungi, at Statens Serum Institut (Boisen et al., 2012). Detection of one of these genes was considered diagnostic of EAEC. Initial screening for EAEC was performed at the Departments of Clinical Microbiology at Slagelse Hospital and Copenhagen University Hvidovre Hospital by PCR targeting the *aggR* gene. EAEC positive strains were forwarded to the Danish National Reference Center at Statens Serum Institut for further characterization as described below. In selected cases, the treating physician had requested microbiological analysis for enteric viruses or parasites to diagnose the cause of diarrhea. Only samples without detection of other pathogens were included in the study.

### Characterization of EAEC strains

Further, characterization of the EAEC strains collected was performed by additional PCR targeting the genes *sat, sepA, pic, sigA, pet, astA, aap, agg3/4C, agg3A, aafA, aggA, agg4A*, and *agg5A* as previously described (Boisen et al., 2012; Jønsson et al., 2015) with modifications. The characterization involved detection of the serine proteases autotransporters of Enterobacteriaceae (SPATEs) including *sat* (secreted autotransporter toxin), *sepA* (*Shigella* extracellular protein), *pic* (serine protease precursor), *sigA* (IgA protease homolog), *pet* (plasmid encoded protein), and the *astA* gene (enteroaggregative *Escherichia coli* heat-stable toxin). Furthermore, included in the characterization were, *aap* (dispersin), ORF3 (open reading frame encoding cryptic protein), and *aggA* (fimbrial subunit for AAF/I), *aafA* (fimbrial subunit for AAF/II), *agg3A* (fimbrial subunit for AAF/III), *agg3/4C* (usher for AAF/III-IV), *agg4A* (fimbrial subunit for AAF/IV), and *agg5A* (fimbrial subunit for AAF/V; Table 1).

### Antimicrobial agent susceptibility testing

Susceptibility toward antibiotics was investigated by using the disk diffusion method according to the Clinical and Laboratory Standards Institute, CLSI, guidelines (Clinical Laboratory Standards Institute, 2011), where 0.5 McFarland standard on Müeller-Hinton II agar plates (BBLTM, US) was used. Susceptibility toward antibiotics was assessed by using the EUCAST breakpoints from the CLSI guidelines (www.EUCAST.org) using the Neo-Sensitabs™ (Rosco Diagnostica A/S, Taastrup, Denmark): gentamicin 10 μg (GEN10), cefotaxime 30 μg (CTX30), cefoxitin 30 μg (CFO30), trimethoprim 5 μg (TRIM5), sulfonamides 240 μg (SULFA), aztreonam 30 μg (AZT30), mecillinam 10 μg (MEC10), tetracycline 30 μg (TET30), chloramphenicol 30 μg (CLR30), nalidixan 30 μg (NAL30), nitrofurantoin 100 μg (NI100), piperacillin 100 μg (PIPRA), and ciprofloxacin 5 μg (CIPR5). Multi-drug resistance was defined as acquired resistance toward three or more antibiotics from different antibiotic classes tested (Magiorakos et al., 2012).

### Questionnaires

When EAEC was the only pathogen detected by the microbiological analysis, the children's parents were contacted by telephone by a medical doctor and were interviewed using a questionnaire. The parents of the children in the EAEC-negative group had answered the same questionnaire. The questionnaire inquired about gastrointestinal symptoms and exposures, including foreign travel, use of antibiotics, and contact with sick animals. In addition, information regarding birth-weight, breastfeeding, infant colic, and pet ownership was inquired.

### Ethics

The study was carried out in accordance with The National Committee on Health Research Ethics with written informed consent from all parents or guardians. All parents or guardians gave written informed consent in accordance with the Declaration of Helsinki and the study was approved by The National Committee on Health Research Ethics, protocol number (H-A-2008-111).

### Statistics

We performed a CART analysis using the Chi-Squared Automatic Interaction Detection (CHAID) growth method with a minimum of five cases for each parent and child node. We used likelihood ratio tests to identify statistically significant branching points between specific EAEC virulence genes and duration of diarrhea. Associations were considered statistically significant, when *p* < 0.05. The *p*-values were Bonferroni corrected to account for multiple testing. We present two CART trees: One where the duration of diarrhea is treated as an interval level construct and one where we treat it as a categorical construct distinguishing between acute diarrhea and persistent diarrhea, respectively. Furthermore, we investigated whether we could find a significant association between EAEC genes and watery diarrhea, bloody diarrhea or mucoid diarrhea.

To identify risk factors associated with EAEC infection, we compared the EAEC-positive group of children with the EAEC-negative group of children. For these analyses, we used independent samples *t*-tests for the interval level constructs, such as age and birth weight, and a difference of proportions test for the categorical constructs, such as gender and pet ownership. In addition, we performed a logistic regression to assess the effect of breastfeeding on the risk of EAEC infection.

Finally, the odds ratios were calculated for each individual EAEC gene in the group of children with acute and persistent diarrhea provided with a 95% confidence interval and *p*-values, by using Fisher's exact test. For data analysis, we used SPSS version 23.0 software for windows (SPSS Inc., USA). The study was approved by the Danish Data Protection Agency, protocol number (2013-41-2338).

## Results

### Annual prevalence of EAEC in children with diarrhea in denmark

To investigate the prevalence of EAEC in childhood diarrhea in Denmark in general, we examined the annual prevalence of all conventional enteropathogens in stool samples submitted for routine microbiological analysis, at the Department of Bacteria, Parasites and Fungi at Statens Serum Institut. In the period from June 2011 until June 2012, a total of 1,360 children aged ≤11 years were examined (Table 4). The percentages of children, who tested positive for bacterial pathogens, were as follows: AEEC (11%; *n* = 146), EAEC (6.7%; *n* = 91), EPEC (2.6%; *n* = 36), *Campylobacter* spp. (2.4%; *n* = 32), *Salmonella* spp. (1.3%; *n* = 18), ETEC (0.7%; *n* = 9), VTEC (0.7%; *n* = 9), *Yersinia* spp. (0.2%; *n* = 2), EIEC (0.1%; *n* = 1), and *Shigella* spp. (0.1%; *n* = 1). During this 1 year period, EAEC was the second most prevalently detected enteric pathogen in Danish children with diarrhea.

**Table 4:** The annual prevalence of bacterial microorganisms in children suffering from diarrhea was investigated at Statens Serum Institut (SSI) in the period between June 2011 until June 2012.

**Microorganism**	**%**	**(No. #)**
**ANNUAL PREVALENCE OF ENTERIC PATHOGENS AT SSI**		
AEEC	11	(146)
EAEC	6.7	(91)
EPEC	2.6	(36)
*Campylobacter* spp.	2.4	(32)
*Salmonella* spp.	1.3	(18)
ETEC	0.7	(9)
VTEC	0.7	(9)
*Yersinia* spp.	0.2	(2)
EIEC	0.1	(1)
*Shigella* spp.	0.1	(1)

### Distribution of EAEC virulence genes

From the 156 EAEC-positive children, 25 children had unknown duration of diarrhea, and in the remaining group of children, 87 EAEC strains were available for subsequent multiplex PCR analysis. The distribution of EAEC virulence genes were *sat* (29%), *sepA* (24%), *pic* (52%), *sigA* (3%), *pet* (10%), *astA* (31%), *aggR* (75%), *aatA* (93%), *aaiC* (67%), *aap* (77%), ORF3 (75%), *agg3/4C* (35%), *agg3A* (7%), *aafA* (7%), *aggA* (16%), *agg4A* (12%), and *agg5A* (20%). The distribution of EAEC genes in children with acute and persistent diarrhea is presented in Table 5.

**Table 5:** The distribution of EAEC genes detected in cases of acute and persistent diarrhea.

**EAEC genes**	**Acute diarrhea *N* = 27 (%)**	**Persistent diarrhea *N* = 45 (%)**	Odds ratio^*^	**Confidence interval**	***P*-value**
*sat*	7 (26)	15 (33)	0.7	(0.22; 1.82)	0.60
*sepA*	7 (26)	10 (22)	1.23	(0.40; 3.72)	0.78
*pic*	21 (78)	18 (40)	5.25	(1.77; 15.45)	0.00
*sigA*	1 (4)	1 (2)	1.69	(0.10; 28.22)	1.00
*pet*	3 (11)	6 (13)	0.81	(0.20; 3.73)	1.00
*astA*	7 (26)	17 (38)	0.58	(0.22; 1.76)	0.44
*aatA*	27 (100)	41 (91)	0.95	(0.38; 1.00)	0.29
*aggR*	26 (96)	32 (71)	10.56	(1.30; 86.15)	0.01
*aaic*	22 (81)	28 (62)	2.67	(0.85; 8.38)	0.12
*aap*	27 (100)	33 (73)	0.73	(0.61; 0.87)	0.00
ORF3	24 (89)	33 (73)	2.91	(0.74; 11.45)	0.14
*agg3/4C*	12 (44)	14 (31)	1.77	(0.66; 4.75)	0.31
*agg3A*	3 (11)	3 (7)	1.75	(0.11;3.06)	0.67
*aafA*	1 (4)	5 (11)	0.31	(0.03; 2.79)	0.40
*aggA*	9 (33)	5 (11)	4	(1.17; 13.64)	0.03
*agg4A*	4 (15)	2 (4)	3.74	(0.64; 21.98)	0.19
*agg5A*	6 (22)	9 (20)	1.14	(0.36; 3.66)	1.00

*The odds ratio was calculated and two-sided p-values were considered statistically significant, when p < 0.05, using Fisher's exact test. The genes aggR, pic, aap, and aggA were individually associated with acute diarrhea, p < 0.05*.

* *When no gene was represented (value = 0) in the categories acute or persistent diarrhea, the relative risk ratio was calculated instead of the odds ratio i.e., for the genes aatA and aap*.

*EAEC, enteroaggregative Escherichia coli; sat, secreted autotransporter toxin; sepA, Shigella extracellular protein; pic, serine protease precursor; sigA, IgA protease homolog; pet, plasmid encoded protein; astA, enteroaggregative Escherichia coli heat-stable toxin; aatA, dispersin transporter protein; aggR, transcription activator; aaiC, secreted protein; aap, dispersin; ORF3, open reading frame encoding cryptic protein; agg3/4C, assembly unit for AAF/III–V; agg3A, fimbrial subunit for AAF/III; aafA, fimbrial subunit for AAF/II; aggA, fimbrial subunit for AAF/I; agg4A, fimbrial subunit for AAF/IV; agg5A, fimbrial subunit for AAF/V*.

### Significance of EAEC virulence genes in disease

**Figure 2:**
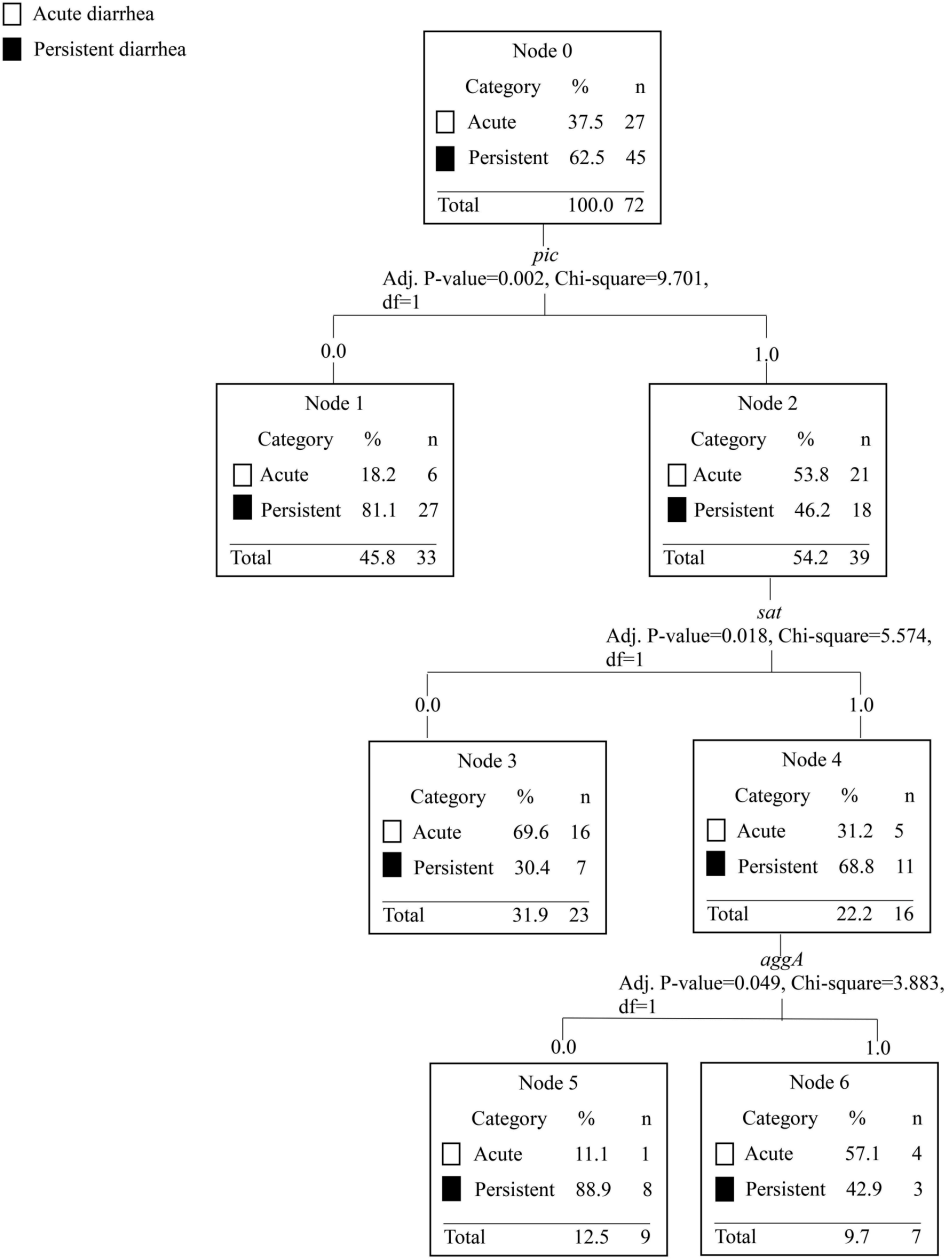
The CART tree analysis shows the affiliations between EAEC genes and acute diarrhea ≤7 days compared with persistent diarrhea ≥ 14 days. *N* is the number of children. The number 0 in each branch indicates the absence of a gene and the number 1 indicates the presence of a gene. The EAEC genes included in the analysis were *sat, sepA, pic, sigA, pet, astA, aap, aaiC, aggR, aatA, ORF3, agg3/4C, agg3A, aafA, aggA, agg4A*, and *agg5A*. Each branch in the CART tree terminates in a “node,” which represents a specific combination of genes, or absence of genes, statistically significant in the categories of acute and persistent diarrhea. Persistent diarrhea was associated with EAEC strains lacking the *pic* gene. EAEC strains possessing the *pic* and *sat* genes, but lacking the *aggA* gene were associated with persistent diarrhea.

**Figure 3:**
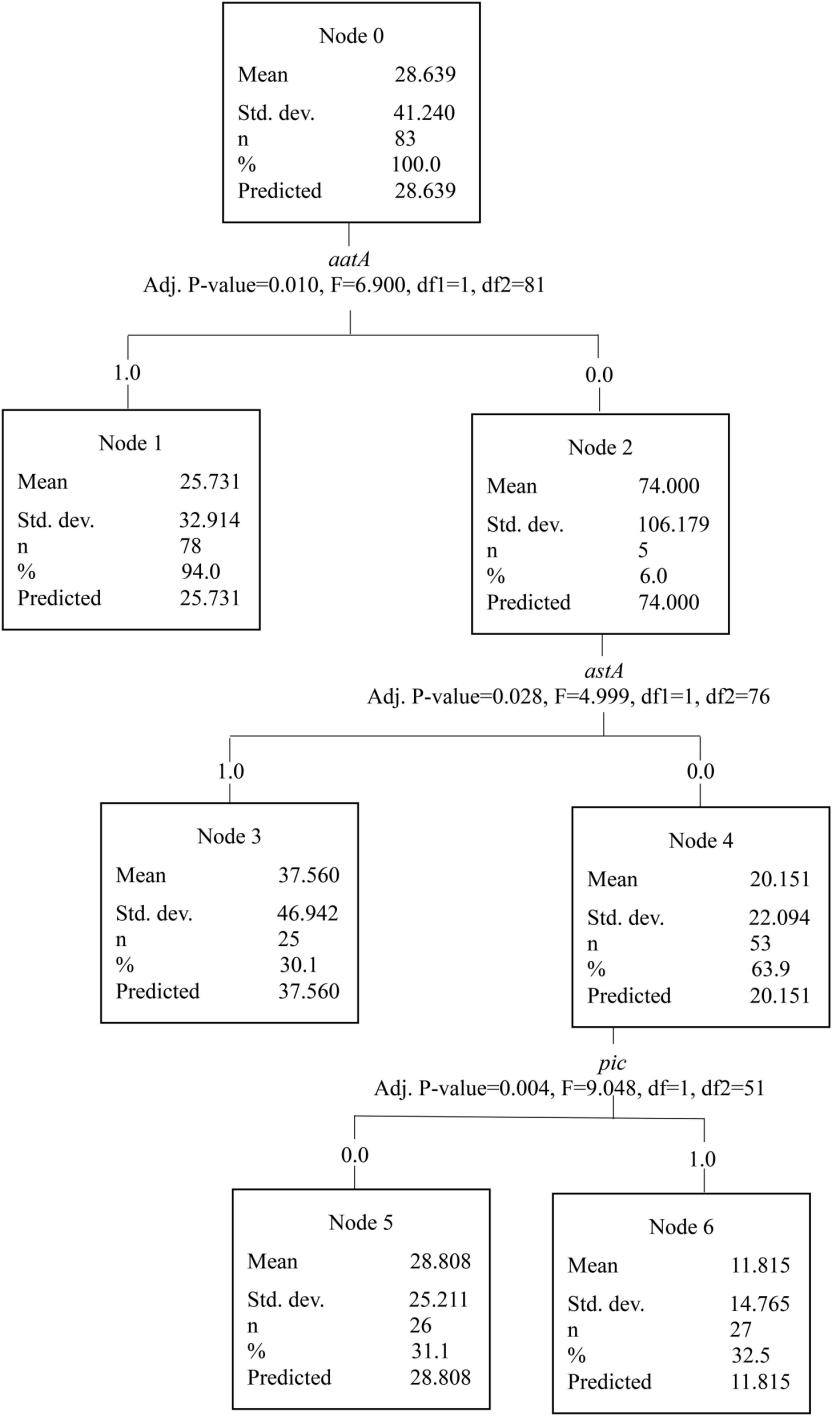
The CART tree analysis shows the affiliations between EAEC genes and duration of diarrhea treated as continuous data. *N* is the number of children. The number 0 in each branch indicates the absence of a gene and the number 1 indicates the presence of a gene. The EAEC genes included in the analysis were *sat, sepA, pic, sigA, pet, astA, aap, aggR, aaiC, aatA, ORF3, agg3*/4C, *agg3A, aafA, aggA, agg4A*, and *agg5*. Each branch in the CART tree terminates in a “node,” which represents a specific set of genes, or absence of genes, with a statistical significant association with different durations of diarrhea in days. Strains lacking the *aatA* gene were associated with diarrhea with the longest median duration (74 days). EAEC strains with the combination of the *aatA* and *astA* genes were associated with prolonged diarrhea with a higher median duration (38 days) compared with EAEC strains lacking the *astA* gene and presence or absence of the *pic* gene (29 and 12 days, respectively).

To assess the association between EAEC genes in acute and persistent diarrhea, we performed a CART tree analysis. This analysis clusters the genes in a stepwise manner according to the investigated categories. With each branch, a statistical significance between the absence and/or presence of a gene further divides the tree into new branches and provides a discrimination between acute and persistent diarrhea, respectively. Five outliers with reports of diarrhea lasting 200 days or longer were removed from the analysis leaving 83 observations. From the 83 observations, 27 EAEC strains were collected from children with acute diarrhea, 45 strains from children with persistent diarrhea. Eleven children had diarrhea lasting 8–13 days. First, we present the results from the CART tree, where we treat duration of diarrhea as a categorical construct using the categories acute and persistent. EAEC strains lacking the *pic* gene were more likely to cause persistent diarrhea (≥14 days), *p* = 0.002 (Figure 2). EAEC strains possessing the genes *pic, sat* but lacking the *aggA* gene were also more likely to cause persistent diarrhea, *p* = 0.05. When duration of diarrhea in days is treated as an interval level construct, we found the absence of the *aatA* gene to be associated with highly prolonged diarrhea (mean duration = 74 days) compared to the grand mean in the sample of 29 days (Figure 3). Presence of the genes *aatA* and *astA* was associated with slightly prolonged diarrhea (mean duration = 38 days; *p* = 0.03).

We calculated the individual odds ratios for EAEC virulence genes in cases of acute and persistent diarrhea. By this analysis, we found the genes *pic, aggR, aap*, and *aggA* to be associated with acute diarrhea (Table 5).

**Figure 4:**
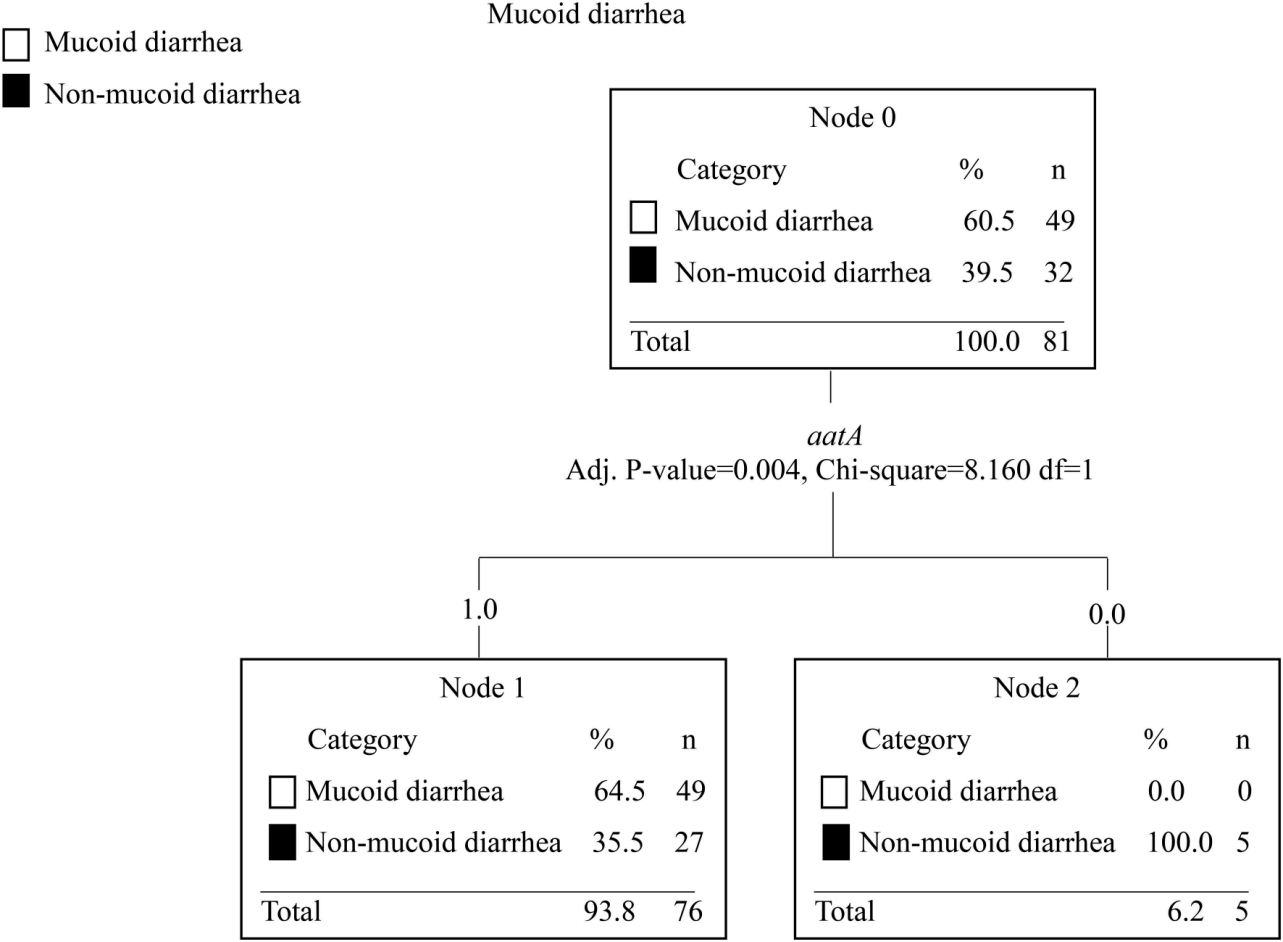
The CART tree analysis shows the association between EAEC genes and mucoid diarrhea. *N* is the number of children. The number 0 in each branch indicates the absence of a gene and the number 1 indicates the presence of a gene. EAEC genes included in the analysis were *sat, sepA, pic, sigA, pet, astA, aap, aggR, aaiC, aatA, ORF3, agg3/4C, agg3A, aafA, aggA, agg4A*, and *agg5*. Each branch in the CART tree terminates in a “node,” which represents a specific set of genes or absence of genes, which are statistically significant in the categories investigated. Strains lacking the *aatA* gene were associated with non-mucoid diarrhea, *p* = 0.004. No association between EAEC genes and watery or bloody diarrhea was observed.

Furthermore, the CART analysis was used to investigate any statistically significant association between EAEC virulence genes and watery, mucoid, and/or bloody diarrhea, respectively. Only the absence of the *aatA* gene was a significant predictor and was associated with *not* having mucoid diarrhea, *p* = 0.004 (Figure 4).

### Clinical manifestations associated with EAEC infection

Parents of children infected with EAEC were interviewed by a medical doctor and symptoms were registered (Table 6). Fever was reported for 35% of the EAEC-positive children and was usually ≤39°C. Other clinical presentations associated with EAEC infection were abdominal cramping (55%), reduced appetite (52%), vomiting (39%), and weight loss (39%). The median weight loss was 1,000 g. Acute diarrhea defined as ≤7 days was reported from 36 (23%) children, but the majority of EAEC-positive children suffered from persistent diarrhea defined as >14 days (*n* = 83, 53%). The duration of diarrhea was 8–13 days for 12 children (8%) and unknown for 25 children (16%). The median duration of EAEC-associated diarrhea was 14 days. The median number of passing diarrheal stools was 6.5 per day at disease maximum. Different categories of diarrhea was reported among EAEC cases, including mixed watery and mucoid diarrhea (*n* = 74, 47%), watery diarrhea (*n* = 56, 36%), mucoid diarrhea only (*n* = 15, 10%), and bloody diarrhea (*n* = 10, 6%).

**Table 6:** Clinical symptoms reported from children infected with EAEC.

**Clinical symptoms reported in EAEC cases (*****n*** = **156)**		
**Symptom**	**(No. #)**	**(%)**
Acute diarrhea (≤7 days)	36	23
Persistent diarrhea (≤14 days)	83	53
Diarrhea lasting 8–13 days	12	8
Unknown duration of diarrhea	25	16
Median duration of diarrhea	14 days	
Median diarrheal stool passage per day at disease maximum	6.5	
Mixed watery and mucoid diarrhea	74	47
Watery diarrhea	56	36
Muciod diarrhea	15	10
Bloody diarrhea	10	6
Fever	55	35
Abdominal cramping	86	55
Reduced appetite	81	52
Vomiting	61	39
Weight loss	61	39
Median weight loss	1,000 g	

### EAEC infection and risk factors

To identify factors associated with an increased risk or protective effect against EAEC infection we compared age, gender, pet-ownership, contact with sick animals, the use of antibiotics, infant colic, and birth-weight between EAEC-positive children and EAEC-negative children. The only statistically significant difference between the two groups was the duration of breastfeeding. The children in the EAEC-negative group had been breastfeed significantly longer compared with the children in the EAEC-positive group (*p* < 0.00; see Table 3). We performed a logistic regression to assess the relationship between EAEC status and the duration of breastfeeding. To illustrate the relationship between duration of breastfeeding and having EAEC infection in a more understandable metric, we graphed the relationship in Figure 4.

### Antibiotic resistance in EAEC strains

Susceptibility testing toward antibiotics was performed for 134 EAEC strains. In general, a high level of resistance toward antibiotics was observed, and multi-drug resistance was seen in 38% (*n* = 51) of the EAEC strains (**Figure 6**). From the multi-drug resistant strains, 35 (69%) were collected from children with reports of foreign travel. Multidrug-resistant EAEC strains were collected from children, who had visited Asia (*n* = 7, 57%, 15 in total), Southern Europe (*n* = 6, 27%, 22 in total), Northern Europe (*n* = 1, 14%, 7 in total), Africa (*n* = 20, 59%, 34 in total) and the Middle East (*n* = 1, 14%, 8 in total). Resistance toward broad-spectrum antibiotics was detected in EAEC strains collected from 14 children with reports of foreign travel, which included resistance against ciprofloxacin in 4 strains and resistance against gentamicin in 10 strains.

### Hospitalization of EAEC infected children

Twenty-three (15%) of the 156 EAEC-positive children with diarrhea were hospitalized. Ten of the hospitalized children were treated with different antibiotics, including broad-spectrum penicillin (*n* = 3), macrolides (*n* = 2), and cefuroxime (*n* = 1). For three children the antibiotics used were unknown. Enteric parasites were examined for in 10 of 23 hospitalized children and enteric viruses in seven hospitalized children. Thirteen hospitalized children (57%) had a history of foreign travel within a period of 2 months prior to examinations, where traveling to Egypt was reported from eight children, while two children had visited Turkey. One child had either visited Ethiopia, Somalia, or Pakistan. The genetic profile of the strains collected from hospitalized children did not differ statistically significantly from other strains (data not shown).

## Discussion

EAEC is an acknowledged common diarrheal pathogen, but the identification of a sole and causative pathogenic EAEC trait remains inconclusive. A large number of virulence factors and combinations hereof, have been associated with clinical illness in epidemiologic studies (Hebbelstrup Jensen et al., 2014). It is plausible that the host immunity and exposure (endemic presence of EAEC) plays an important role in EAEC pathogenicity, therefore, the combination of virulence genes in EAEC provides the necessary variety for local disease. The mosaic nature of EAEC genomes described today seems to enhance our idea that EAEC pathogenicity is very dependent on the host and its environment.

In this study, we have characterized a number of EAEC strains collected from children with acute and persistent diarrhea. The EAEC strains were characterized in respect of classical EAEC virulence genes and antibiotic resistance profiles. We found the absence of the *pic* gene to be associated with persistent diarrhea, *p* = 0.02. On the other hand, the combination of the genes *pic* and *sat* and absence of the *aggA* gene was associated with persistent diarrhea, *p* = 0.05 (Figure 2). The *pic* gene has mucinolytic activity, it causes hemagglutination and serum resistance (Henderson et al., 1999). Pic is a protease, which is secreted by EAEC and *Shigella flexneri* (Henderson et al., 1999) and it has been shown to play a key role in the colonization and growth of EAEC in mucus in a mouse model (Harrington et al., 2009). The combination of the genes *pic, sepA*, and *agg4A* has been associated with the formation of strong biofilm (Nezarieh et al., 2015). Biofilm formation is a key pathogenic trait for the development of persistent infection (Costerton et al., 1999) and the formation of biofilm is what separates EAEC from other diarrheagenic *E. coli* pathotypes. The *sat* gene has been shown to cause intestinal damage with fluid accumulation and villus necrosis in a rabbit ileal loop model (Taddei et al., 2005). *Sat* was one of the most prevalent genes detected in children with diarrhea in an Iranian study (Nezarieh et al., 2015). The *aggA* gene encodes a subunit of the aggregative fimbria type I, which has been shown to elicit an immunogenic response in a mouse vaccination study (Bouzari et al., 2010). It could be speculated that the children were partly protected from antibodies toward the aggregative fimbria type I, due to previous exposure resulting in a shortened span of disease.

EAEC strains with the combination of the *aatA* and *astA* genes were associated with prolonged diarrhea (*p* = 0.03; Figure 3). The *aatA* gene is a key EAEC gene and it encodes part of an outer membrane transport system involved in translocation of the dispersin protein. The *aatA* gene corresponds to a part of the EAEC virulence plasmid pCVD432 (Baudry et al., 1990). It was one of the first genes targeted by PCR to diagnose EAEC (Nishi et al., 2003). The genes *astA* and *pic* are both present in the prototype EAEC strain 042, which has been associated with diarrhea in a volunteer study (Nataro et al., 1995). The *aatA* gene has been associated with the formation of biofilm (Mohamed et al., 2007), which is characterized by a thick mucus layer in the intestine and predisposes to persistent infection (Costerton et al., 1999). The *astA* gene encodes the EAST-1 toxin, and it was one of the first virulence factors associated with diarrheagenic EAEC strains (Ménard and Dubreuil, 2002), but *astA* is not restricted to EAEC (Savarino et al., 1996). The EAST-1 toxin has been shown to elicit a secretory response in a rabbit ileal model (Savarino et al., 1991). It could be speculated that this response induces an inflammatory response with protracted disease manifestations. Thus, in combination, the *aatA* and *astA* genes could be responsible for prolonged inflammation and formation of biofilm in the intestine resulting in prolonged diarrhea.

Strains lacking the *aatA* gene were associated with non-mucoid diarrhea, *p* = 0.00 (Figure 4). A wide range of EAEC genes have been associated with increased secretion of mucus, such as *pic* (Navarro-Garcia et al., 2010) and *pet* (Navarro-García et al., 1998). The combination of the *aatA, aggR, astA*, and *aap* genes has been associated with gross mucus and leukocytes in stools from patients with diarrhea compared with healthy controls, *p* < 0.05 (Cennimo et al., 2009). Collectively, our results suggest that the pathophysiology of EAEC enteric infection involves a complex and dynamic modulation of several virulence genes.

The CART analysis suggested the *sat* and *astA* genes for virulent EAEC strains with persistent disease manifestations. Strains, with the *aggA* gene in different combinations with other EAEC genes were associated with less-pathogenic EAEC strains, with shorter duration of disease. Suggesting that the fimbriae themselves might not be the sole cause for disease. The odds ratio for the individual EAEC genes between acute and persistent diarrhea suggested an association between the genes *aap, aggR, pic* and *aggA*, and acute diarrhea (Table 5).

In two other studies where the CART analysis was applied (Boisen et al., 2012; Lima et al., 2013), it was found that virulent EAEC strains in Mali harbored the EAEC heat-stable toxin 1 (EAST-1 enterotoxin), and the flagellar type H33 correlated with diarrhea. A Brazilian study (Lima et al., 2013), identified trait clusters in EAEC strains (isolated genes or in combination), which correlated with both children with diarrhea (*pet* and *aafA*) and healthy children (*agg4A* and ORF61). In Ghana, it was found that the presence of the *aap* gene was significantly associated with diarrhea, even though the *aatA* was the most prevalent gene among the EAEC isolates tested (Opintan et al., 2010). These findings suggest that EAEC infection involves a complex and dynamic modulation of several virulence genes for the bacteria in combination with its endemic presence in the population and the immunity present in the population.

The variation of the EAEC genetic repertoire in relation to disease determined in Danish strains, as well as from different populations of distinct geographical regions of the world, confirms again the genetic heterogeneity of EAEC (Huppertz et al., 1997; Glandt et al., 1999; Sarantuya et al., 2004; Huang et al., 2007; Oundo et al., 2008). Furthermore, it is very possible that since many of the virulence genes and antibiotic resistance genes are encoded on plasmids, the dynamic horizontal acquisition and loss of genetic traits can be explained by the favoring of the variety of genetic profiles found in EAEC strains.

**Figure 5:**
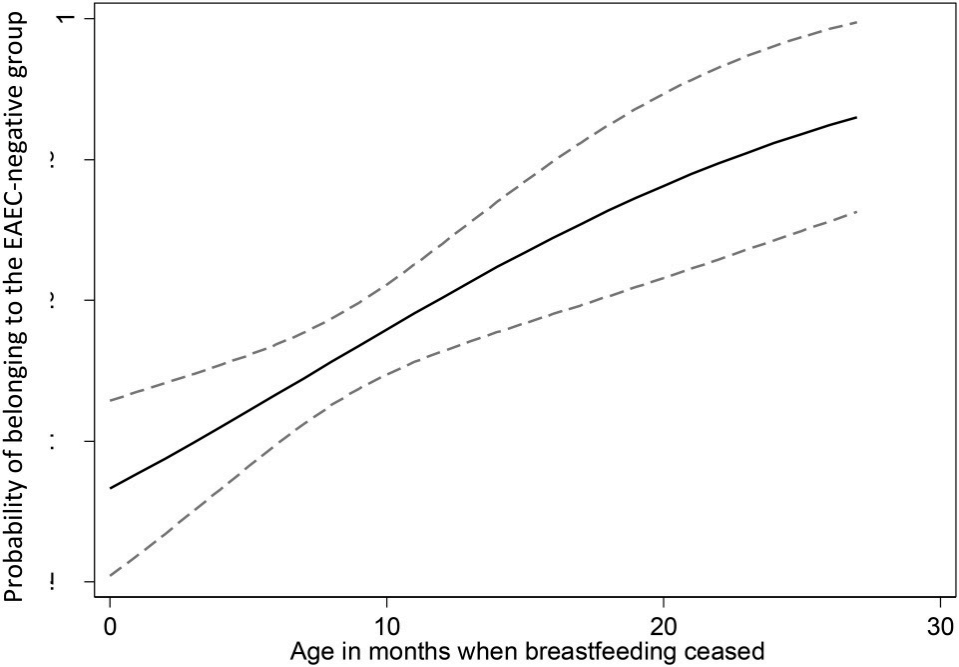
The duration of breastfeeding was positively correlated with the likelihood of belonging to the EAEC-negative group of children with 95% confidence intervals.

In order to investigate general predisposing factors associated with EAEC infection, we compared children with and without EAEC. Previous studies have shown that infant colic predisposes to gastrointestinal diseases later in life (Collado et al., 2012). Although EAEC is not generally considered to be transmitted through animals it has been isolated from animals (Puño-Sarmiento et al., 2013), and we investigated if having a pet could pose a risk for EAEC infection. None of the factors investigated were represented more frequently in the EAEC-positive group, however, prolonged breastfeeding was discovered to be strongly associated with the EAEC-negative group of children (Table 3 and Figure 5). It is known that breastfeeding is protective toward infectious diarrhea (Mølbak et al., 1994). Here, we observed a protective effect of breastfeeding against EAEC infection beyond the age of weaning. Only three (2%) children in the EAEC-negative group and nine (6%) in the EAEC-positive group were still breastfed while included in the study (Table 3). In the period prior to inclusion in the study, more children had been breastfed in the EAEC-negative group compared to the EAEC-positive group (94 vs. 80%). Immunological components in the breast milk such as immunoglobulins, lactoferrin, and lymphocytes have been shown to be crucial for the development of the immune system (Hanson, 1999). Furthermore, benefits in term of long-term protection against infections have previously been observed with prolonged breastfeeding (Hanson, 2004). A health bias in the EAEC negative group could be speculated to effect the analysis since resourceful parents may be more likely to attend such a study. On the other hand, the groups were very comparable in many other aspects (Table 3), and the children were recruited within the same geographical area and in the same period in time.

Examination for enteric viruses was performed for 23 (15%) of the EAEC-positive children. It has been shown, that enteric viruses are highly prevalent in diarrheal episodes in this age group (Olesen et al., 2005) and this could be a confounding factor in the children in our study, who lacked examinations for enteric viruses. However, gastrointestinal viruses often causes acute diarrhea with a high diarrheal output, which was not the clinical manifestations of disease in the majority of children in this study (Table 6). Enteric parasites were examined for in 53 (34%) of the EAEC-positive children. Parasitic infection is mostly associated with traveler's diarrhea in Denmark, which 51% of the children in this study suffered from. Therefore, parasitic infection may be a confounding factor for the 17% of the children with reports of travel in this study.

Hospitalization was reported for 23 (15%) of the EAEC-positive children and was mainly seen in children at the age of ≤2 years. It is well-known, that very young children are more susceptible to dehydration caused by infectious diarrhea (Ramaswamy and Jacobson, 2001), and it has been described for other diarrheagenic *E. coli* pathotypes, such as EPEC (Essers et al., 2000). We did not discover any particular EAEC virulence gene to be associated with hospitalization.

The EAEC-positive children had more reports of foreign travel within a period of 2 months prior to examination, when compared to the EAEC-negative group of children (51 vs. 16%; Table 3). However, the majority of the included children were recruited from Copenhagen University Hvidovre Hospital, where only patients suffering from travelers' diarrhea were investigated for EAEC (Figure 1). EAEC was diagnosed in all categories of diarrhea at SSI, and a total of 48 children were included from this site. From the 48 children diagnosed at SSI, 14 (29%) had reports of foreign travel. Other studies have shown a strong association between foreign travel and EAEC-associated diarrhea in children (Huppertz et al., 1997; Denno et al., 2012).

**Figure 6:**
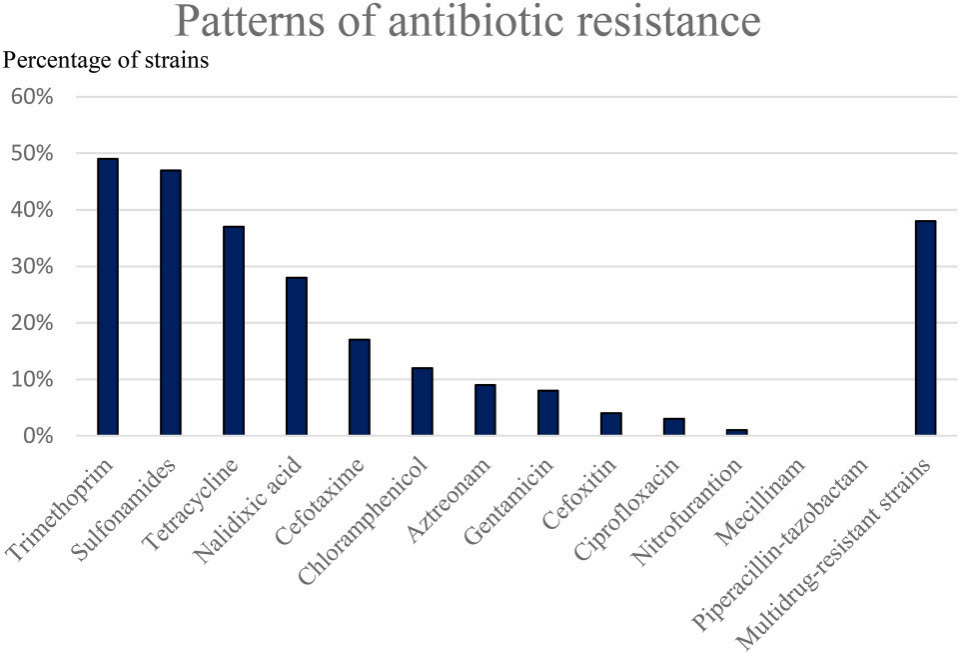
Susceptibility toward antibiotics was tested in the EAEC strains by using the disc diffusion method. A high level of resistance was observed toward trimethoprim, sulfonamides, tetracycline, nalidixic acid, and to a lesser extent, cefotaxime and chloramphenicol.

We detected a high level of antibiotic resistance among the EAEC strains, which considerably limited the treatment possibilities in diarrheal cases. Resistance toward gentamicin was observed in 10 (7.5%) of the EAEC strains and resistance toward ciprofloxacin in 4 (3%) of the EAEC strains (Figure 6). An Indian study showed a high level of resistance toward ciprofloxacin in 63.4% (*n* = 40) of strains tested (Raju and Ballal, 2009) this phenomenon is mostly described in cases of travelers' diarrhea (Vila et al., 2001). Gentamicin resistance in EAEC strains is rarely reported and only in low levels (Khoshvaght et al., 2014; Hebbelstrup Jensen et al., 2016b). Multidrug-resistance was most frequently detected in cases of travelers' diarrhea (69 vs. 31%). Yet, multidrug-resistance in EAEC strains is reported in several other studies (Sang et al., 1997; Raju and Ballal, 2009; Aslani et al., 2011; Hebbelstrup Jensen et al., 2016b) in both industrialized and developing countries, which is a major cause for concern. However, the efficacy of antibiotics in the treatment of EAEC infection remains to be determined and it should be restricted to the severe cases only. The vast majority of EAEC gastrointestinal infections should be treated with fluid and electrolyte replacement similar to infectious diarrhea in general. However, all strains in this study were susceptible to mecillinam and piperacillin-tazobactam, which must be regarded as the drugs of choice in the few selected cases of EAEC-associated diarrhea that requires treatment.

## Conclusion

Persistent diarrhea was associated with EAEC strains without the *pic* gene, and with strains with the combination of the genes *pic* and *sat* and absence of the *aggA* gene. The combination of the *aatA* and *astA* genes was associated with prolonged diarrhea. Acute diarrhea was associated with the genes *aggR, aap*, and *aagA* by individual odds ratios. Strains lacking the *aatA* gene were associated with non-mucoid diarrhea. Breastfeeding was seen to be protective against EAEC infection beyond the age of weaning.

## Author contributions

KK, AMP, JE acquired the funding for this research. BH and AP performed the interviews of participating parents. SH performed the statistical analysis and performed critical appraisal. CS, RJ, AMP, NB, JE and RP were responsible for the microbial analysis performed. BH, CS, NB, RJ were responsible for the interpretation of the microbial analysis performed. All authors contributed to the writing of this manuscript and all authors approved of the final draft. All authors take responsibility for the integrity and the accuracy of this research and the interpretation hereof.

### Conflict of interest statement

The authors declare that the research was conducted in the absence of any commercial or financial relationships that could be construed as a potential conflict of interest.

## References

[B1] Albert M. J. Faruque A. S. Faruque S. M. Sack R. B. Mahalanabis D. (1999). Case-control study of enteropathogens associated with childhood diarrhea in Dhaka, Bangladesh. J. Clin. Microbiol. 37, 3458–3464. 10523534 10.1128/jcm.37.11.3458-3464.1999 PMC85667

[B2] Aslani M. M. Alikhani M. Y. Zavari A. Yousefi R. Zamani A. R. (2011). Characterization of enteroaggregative *Escherichia coli* (EAEC) clinical isolates and their antibiotic resistance pattern. Int. J. Infect. Dis. 15, e136–e139. 10.1016/j.ijid.2010.10.002 21130676

[B3] Baudry B. Savarino S. J. Vial P. Kaper J. B. Levine M. M. (1990). A sensitive and specific DNA probe to identify enteroaggregative *Escherichia coli*, a recently discovered diarrheal pathogen. J. Infect. Dis. 161, 1249–1251. 10.1093/infdis/161.6.1249 2189007

[B4] Bhan M. K. Raj P. Levine M. M. Kaper J. B. Bhandari N. Srivastava R.. (1989). Enteroaggregative *Escherichia coli* associated with persistent diarrhea in a cohort of rural children in India. J. Infect. Dis. 159, 1061–1064. 10.1093/infdis/159.6.1061 2656875

[B5] Blom M. Meyer A. Gerner-Smidt P. Gaarslev K. Espersen F. (1999). Evaluation of Statens Serum Institut enteric medium for detection of enteric pathogens. J. Clin. Microbiol. 37, 2312–2316. 10364603 10.1128/jcm.37.7.2312-2316.1999 PMC85145

[B6] Boisen N. Scheutz F. Rasko D. A. Redman J. C. Persson S. Simon J.. (2012). Genomic characterization of enteroaggregative *Escherichia coli* from children in Mali. J. Infect. Dis. 205, 431–444. 10.1093/infdis/jir757 22184729 PMC3256949

[B7] Bouzari S. Dashti A. Jafari A. Oloomi M. (2010). Immune response against adhesins of enteroaggregative *Escherichia coli* immunized by three different vaccination strategies (DNA/DNA, Protein/Protein, and DNA/Protein) in mice. Comp. Immunol. Microbiol. Infect. Dis. 33, 215–225. 10.1016/j.cimid.2008.10.002 19022502

[B8] Cennimo D. Abbas A. Huang D. B. Chiang T. (2009). The prevalence and virulence characteristics of enteroaggregative Escherichia coli at an urgent-care clinic in the USA: a case-control study. J. Med. Microbiol. 58, 403–407. 10.1099/jmm.0.005793-0 19273633

[B9] Clinical and Laboratory Standards Institute CLSI (2011). Performance Standards for Antimicrobial Susceptibility Testing; Twenty-First Informational Supplements. M100-S21. 31 No. 1. Wayne, PA: Clinical and Laboratory Standards Institute.

[B10] Cobeljić M. Miljković-Selimović B. Paunović-Todosijević D. Velicković Z. Lepsanović Z. Zec N.. (1996). Enteroaggregative *Escherichia coli* associated with an outbreak of diarrhoea in a neonatal nursery ward. Epidemiol. Infect. 117, 11–16. 10.1017/S0950268800001072 8760945 PMC2271665

[B11] Collado M. C. Cernada M. Baüerl C. Vento M. Pérez-Martínez G. (2012). Microbial ecology and host-microbiota interactions during early life stages. Gut Microbes 3, 352–365. 10.4161/gmic.21215 22743759 PMC3463493

[B12] Costerton J. W. Stewart P. S. Greenberg E. P. (1999). Bacterial biofilms: a common cause of persistent infections. Science 284, 1318–1322. 10.1126/science.284.5418.1318 10334980

[B13] Czeczulin J. R. Balepur S. Hicks S. Phillips A. Hall R. Kothary M. H. (1997). Aggregative adherence fimbria II, a second fimbrial antigen mediating aggregative adherence in enteroaggregative *Escherichia coli*. Infect. Immun. 65, 4135–4145. 10.1128/iai.65.10.4135-4145.1997 PMC175595 9317019

[B14] Denno D. M. Shaikh N. Stapp J. R. Qin X. Hutter C. M. Hoffman V.. (2012). Diarrhea etiology in a pediatric emergency department: a case control study. Clin. Infect. Dis. 55, 897–904. 10.1093/cid/cis553 22700832 PMC3657524

[B15] Dudley E. G. Thomson N. R. Parkhill J. Morin N. P. Nataro J. P. (2006). Proteomic and microarray characterization of the AggR regulon identifies a pheU pathogenicity island in enteroaggregative *Escherichia coli*. Mol. Microbiol. 61, 1267–1282. 10.1111/j.1365-2958.2006.05281.x 16925558

[B16] Eslava C. Navarro-García F. Czeczulin J. R. Henderson I. R. Cravioto A. Nataro J. P. (1998). Pet, an autotransporter enterotoxin from enteroaggregative *Escherichia coli*. Infect. Immun. 66, 3155–3163. 9632580 10.1128/iai.66.7.3155-3163.1998 PMC108327

[B17] Essers B. Burnens A. P. Lanfranchini F. M. Somaruga S. G. von Vigier R. O. Schaad U. B. (2000). Acute community-acquired diarrhea requiring hospital admission in Swiss children. Clin. Infect. Dis. 31, 192–196. 10.1086/313901 10913424

[B18] Glandt M. Adachi J. A. Mathewson J. J. Jiang Z. D. DiCesare D. Ashley D.. (1999). Enteroaggregative *Escherichia coli* as a cause of traveler's diarrhea: clinical response to ciprofloxacin. Clin. Infect. Dis. 29, 335–338. 10.1086/520211 10476738

[B19] Guiral E. Mendez-Arancibia E. Soto S. M. Salvador P. Fabrega A. Gascon J.. (2011). CTX-M-15-producing enteroaggregative *Escherichia coli* as cause of travelers' diarrhea. Emerging Infect. Dis. 17, 1950–1953. 10.3201/eid1710.110022 22000380 PMC3310664

[B20] Hanson L. A. (1999). Human milk and host defence: immediate and long-term effects. Acta Paediatr Suppl. 88, 42–46. 10.1111/j.1651-2227.1999.tb01299.x 10569222

[B21] Hanson L. A. (2004). Protective effects of breastfeeding against urinary tract infection. Acta Paediatr. 93, 154–156. 10.1111/j.1651-2227.2004.tb00695.x 15046263

[B22] Harada T. Hiroi M. Kawamori F. Furusawa A. Ohata K. Sugiyama K.. (2007). A food poisoning diarrhea outbreak caused by enteroaggregative *Escherichia coli* serogroup O126:H27 in Shizuoka, Japan. Jpn. J. Infect. Dis. 60, 154–155. Available online at: http://www0.nih.go.jp/JJID/60/154.pdf 17515660

[B23] Harrington S. M. Sheikh J. Henderson I. R. Ruiz-Perez F. Cohen P. S. Nataro J. P. (2009). The pic protease of enteroaggregative *Escherichia coli* promotes intestinal colonization and growth in the presence of mucin. Infect. Immun. 77, 2465–2473. 10.1128/IAI.01494-08 19349428 PMC2687332

[B24] Hebbelstrup Jensen B. Olsen K. E. Struve C. Krogfelt K. A. Petersen A. M. (2014). Epidemiology and clinical manifestations of enteroaggregative *Escherichia coli*. Clin. Microbiol. Rev. 27, 614–630. 10.1128/CMR.00112-13 24982324 PMC4135892

[B25] Hebbelstrup Jensen B. Röser D. Andreassen B. U. Olsen K. E. Nielsen H. V. Roldgaard B. B.. (2016a). Childhood diarrhoea in Danish day care centres could be associated with infant colic, low birth weight and antibiotics. Acta Paediatr. 105, 90–95. 10.1111/apa.13209 26355526

[B26] Hebbelstrup Jensen B. Stensvold C. R. Struve C. Olsen K. E. Scheutz F. Boisen N.. (2016b). Enteroaggregative *Escherichia coli* in daycare - A 1-year dynamic cohort study. Front. Cell. Infect. Microbiol. 6:75. 10.3389/fcimb.2016.00075 27468409 PMC4942469

[B27] Henderson I. R. Czeczulin J. Eslava C. Noriega F. Nataro J. P. (1999). Characterization of pic, a secreted protease of *Shigella flexneri* and enteroaggregative *Escherichia coli. Infect*. Immun. 67, 5587–5596. 10.1128/iai.67.11.5587-5596.1999 PMC96930 10531204

[B28] Hicks S. Candy D. C. Phillips A. D. (1996). Adhesion of enteroaggregative *Escherichia coli* to pediatric intestinal mucosa *in vitro*. Infect. Immun. 64, 4751–4760. 8890236 10.1128/iai.64.11.4751-4760.1996 PMC174442

[B29] Huang D. B. Mohamed J. A. Nataro J. P. DuPont H. L. Jiang Z. D. Okhuysen P. C. (2007). Virulence characteristics and the molecular epidemiology of enteroaggregative *Escherichia coli* isolates from travellers to developing countries. J. Med. Microbiol. 56, 1386–1392. 10.1099/jmm.0.47161-0 17893178

[B30] Huppertz H. I. Rutkowski S. Aleksic S. Karch H. (1997). Acute and chronic diarrhoea and abdominal colic associated with enteroaggregative *Escherichia coli* in young children living in western Europe. Lancet 349, 1660–1662. 10.1016/S0140-6736(96)12485-5 9186384

[B31] Hutchinson D. N. Bolton F. J. (1984). Improved blood free selective medium for the isolation of *Campylobacter jejuni* from faecal specimens. J. Clin. Pathol. 37, 956–957. 10.1136/jcp.37.8.956-b 6381549 PMC498901

[B32] Jenkins C. Chart H. Willshaw G. A. Cheasty T. Tompkins D. S. (2007). Association of putative pathogenicity genes with adherence characteristics and fimbrial genotypes in typical enteroaggregative *Escherichia coli* from patients with and without diarrhoea in the United Kingdom. Eur. J. Clin. Microbiol. Infect. Dis. 26, 901–906. 10.1007/s10096-007-0388-z 17899229

[B33] Jenkins C. Tembo M. Chart H. Cheasty T. Willshaw G. A. Phillips A. D.. (2006). Detection of enteroaggregative *Escherichia coli* in faecal samples from patients in the community with diarrhoea. J. Med. Microbiol. 55, 1493–1497. 10.1099/jmm.0.46683-0 17030907

[B34] Jiang Z. D. Okhuysen P. C. Guo D. C. He R. King T. M. DuPont H. L. (2003). Genetic susceptibility to enteroaggregative *Escherichia coli* diarrhea: polymorphism in the interleukin-8 promotor region. J. Infect. Dis. 15, 506–511. 10.1086/377102 12898436

[B35] Jokelainen P. Hebbelstrup Jensen B. Andreassen B. U. Petersen A. M. Röser D. Krogfelt K. A.. (2017). *Dientamoeba fragilis*- a commensal in children in Danish day care centers. J. Clin. Microbiol. 55, 1707–1713. 10.1128/JCM.00037-17 28330885 PMC5442526

[B36] Jønsson R. Struve C. Boisen N. Mateiu R. V. Santiago A. E. Jenssen H.. (2015). Novel aggregative adherence fimbria variant of enteroaggregative *Escherichia coli*. Infect. Immun. 83, 1396–1405. 10.1128/IAI.02820-14 25624357 PMC4363450

[B37] Khoshvaght H. Haghi F. Zeighami H. (2014). Extended spectrum betalactamase producing Enteroaggregative *Escherichia coli* from young children in Iran. *Gastroenterol. Hepatol*. Bed. Bench. 7, 131–136. PMC4017568 24834305

[B38] Lima A. A. Moore S. R. Barboza M. S. Jr Soares A. M. Schleupner M. A. Newman R. D.. (2000). Persistent diarrhea signals a critical period of increased diarrhea burdens and nutritional shortfalls: a prospective cohort study among children in northeastern Brazil. J. Infect. Dis. 181, 1643–1651. 10.1086/315423 10823764

[B39] Lima I. F. Boisen N. Quetz J.da S. Havt A. de Carvalho E. B. Soares A. M.. (2013). Prevalence of enteroaggregative *Escherichia coli* and its virulence-related genes in a case-control study among children from north-eastern Brazil. J. Med. Microbiol. 62, 683–693. 10.1099/jmm.0.054262-0 23429698 PMC3709657

[B40] Magiorakos A. P. Srinivasan A. Carey R. B. Carmeli Y. Falagas M. E. Giske C. G.. (2012). Multidrug-resistant, extensively drug-resistant and pandrug-resistant bacteria: an international expert proposal for interim standard definitions for acquired resistance. Clin. Microbiol. Infect. 18, 268–281. 10.1111/j.1469-0691.2011.03570.x 21793988

[B41] Medina A. M. Rivera F. P. Romero L. M. Kolevic L. A. Castillo M. E. Verne E.. (2010). Diarrheagenic *Escherichia coli* in human immunodeficiency virus (HIV) pediatric patients in Lima, Peru. Am. J. Trop. Med. Hyg. 83, 158–163. 10.4269/ajtmh.2010.09-0596 20595495 PMC2912593

[B42] Ménard L. P. Dubreuil J. D. (2002). Enteroaggregative *Escherichia coli* heat-stable enterotoxin 1 (EAST1): a new toxin with an old twist. Crit. Rev. Microbiol. 28, 43–60. 10.1080/1040-840291046687 12003040

[B43] Mendez-Arancibia E. Vargas M. Soto S. Ruiz J. Kahigwa E. Schellenberg D.. (2008). Prevalence of different virulence factors and biofilm production in enteroaggregative *Escherichia coli* isolates causing diarrhea in children in Ifakara (Tanzania). Am. J. Trop. Med. Hyg. 78, 985–989. Available online at: http://www.ajtmh.org/content/78/6/985.long 18541781

[B44] Mohamed J. A. DuPont H. L. Flores J. Palur H. Nair P. Jiang Z. D.. (2011). Single nucleotide polymorphisms in the promoter of the gene encoding the lipopolysaccharide receptor CD14 are associated with bacterial diarrhea in US and Canadian travelers to Mexico. Clin. Infect. Dis. 52, 1332–1341. 10.1093/cid/cir228 21596674 PMC3140174

[B45] Mohamed J. A. Huang D. B. Jiang Z. D. DuPont H. L. Nataro J. P. Belkind-Gerson J.. (2007). Association of putative enteroaggregative *Escherichia coli* virulence genes and biofilm production in isolates from travelers to developing countries. J. Clin. Microbiol. 45, 121–126. 10.1128/JCM.01128-06 17093030 PMC1828990

[B46] Mølbak K. Gottschau A. Aaby P. Højlyng N. Ingholt L. da Silva A. P. (1994). Prolonged breast feeding, diarrhoeal disease, and survival of children in Guinea-Bissau. BMJ 28:308 10.1136/bmj.308.6941.1403 PMC2540341 8019249

[B47] Morin N. Santiago A. E. Ernst R. K. Guillot S. J. Nataro J. P. (2013). Characterization of the AggR regulon in enteroaggregative *Escherichia coli*. Infect. Immun. 81, 122–132. 10.1128/IAI.00676-12 23090962 PMC3536136

[B48] Nataro J. P. Deng Y. Cookson S. Cravioto A. Savarino S. J. Guers L. D.. (1995). Heterogeneity of enteroaggregative *Escherichia coli* virulence demonstrated in volunteers. J. Infect. Dis. 171, 465–468. 10.1093/infdis/171.2.465 7844392

[B49] Navarro-García F. Eslava C. Villaseca J. M. López-Revilla R. Czeczulin J. R. Srinivas S.. (1998). *In vitro* effects of a high-molecular-weight heat-labile enterotoxin from enteroaggregative *Escherichia coli*. Infect. Immun. 66, 3149–3154. 9632579 10.1128/iai.66.7.3149-3154.1998 PMC108326

[B50] Navarro-Garcia F. Gutierrez-Jimenez J. Garcia-Tovar C. Castro L. A. Salazar-Gonzalez H. Cordova V. (2010). Pic, an autotransporter protein secreted by different pathogens in the Enterobacteriaceae family, is a potent mucus secretagogue. Infect. Immun. 78, 4101–4109. 10.1128/IAI.00523-10 20696826 PMC2950354

[B51] Nezarieh R. Shakibaie M. R. Nave H. H. Norouzi A. Salajegheh G. Hayatbakhsh M. (2015). Distribution of virulence genes, enterotoxin and biofilm formation among enteroaggregative *Escherichia coli* (EAEC) strains isolated from stools of children with diarrhea in South East Iran. Arch. Ped. Infec. Dis. 3:e29745 10.5812/pedinfect.29745v2

[B52] Nishi J. Sheikh J. Mizuguchi K. Luisi B. Burland V. Boutin A.. (2003). The export of coat protein from enteroaggregative *Escherichia coli* by a specific ATP-binding cassette transporter system. J. Biol. Chem. 278, 45680–45689 10.1074/jbc.m306413200 12933818

[B53] Olesen B. Neimann J. Böttiger B. Ethelberg S. Schiellerup P. Jensen C.. (2005). Etiology of diarrhea in young children in Denmark: a case-control study. J. Clin. Microbiol. 43, 3636–3641. 10.1128/JCM.43.8.3636-3641.2005 16081890 PMC1234006

[B54] Opintan J. A. Newman M. J. Ayeh-Kumi P. F. Affrim R. Gepi-Attee R. Sevilleja J. E.. (2010). Pediatric diarrhea in southern Ghana: etiology and association with intestinal inflammation and malnutrition. Am. J. Trop. Med. Hyg. 83, 936–943. 10.4269/ajtmh.2010.09-0792 20889896 PMC2946773

[B55] Oundo J. O. Kariuki S. M. Boga H. I. Muli F. W. Iijima Y. (2008). High incidence of enteroaggregative *Escherichia coli* among food handlers in three areas of Kenya: a possible transmission route of travelers' diarrhea. J. Travel Med. 15, 31–38. 10.1111/j.1708-8305.2007.00174.x 18217867

[B56] Pereira A. L. Ferraz L. R. Silva R. S. Giugliano L. G. (2007). Enteroaggregative *Escherichia coli* virulence markers: positive association with distinct clinical characteristics and segregation into 3 enteropathogenic *E. coli* serogroups. J. Infect. Dis. 195, 366–374. 10.1086/510538 17205475

[B57] Persson S. Al-Shuweli S. Yapici S. Jensen J. N. Olsen K. E. (2015). Identification of clinical Aeromonas Species by rpoB and gyrB sequencing and development of a multiplex PCR method for detection of *Aeromonas hydrophila, A. caviae, A. veronii*, and *A. media*. J. Clin. Microbiol. 53, 653–656. 10.1128/JCM.01963-14 25411168 PMC4298543

[B58] Persson S. Olsen K. E. Scheutz F. Krogfelt K. A. Gerner-Smidt P. (2007). A method for fast and simple detection of major diarrhoeagenic *Escherichia coli* in the routine diagnostic laboratory. Clin. Microbiol. Infect. 13, 516–524. 10.1111/j.1469-0691.2007.01692.x 17331124

[B59] Persson S. Torpdahl M. Olsen K. E. (2008). New multiplex PCR method for the detection of Clostridium difficile toxin A (tcdA) and toxin B (tcdB) and the binary toxin (cdtA/cdtB) genes applied to a Danish strain collection. Clin. Microbiol. Infect. 14, 1057–1064. 10.1111/j.1469-0691.2008.02092.x 19040478

[B60] Puño-Sarmiento J. Medeiros L. Chiconi C. Martins F. Pelayo J. Rocha S.. (2013). Detection of diarrheagenic *Escherichia coli* strains isolated from dogs and cats in Brazil. Vet. Microbiol. 166, 676–680. 10.1016/j.vetmic.2013.07.007 23932311

[B61] Raju B. Ballal M. (2009). Multidrug resistant enteroaggregative *Escherichia coli* diarrhoea in rural southern Indian population. Scand. J. Infect. Dis. 41, 105–108. 10.1080/00365540802641856 19107675

[B62] Ramaswamy K. Jacobson K. (2001). Infectious diarrhea in children. Gastroenterol. Clin. North. Am. 30, 611–624. 10.1016/S0889-8553(05)70201-6 11586548

[B63] Sang W. K. Oundo J. O. Mwituria J. K. Waiyaki P. G. Yoh M. Iida T.. (1997). Multidrug-resistant enteroaggregative *Escherichia coli* associated with persistent diarrhea in Kenyan children. Emerging Infect. Dis. 3, 373–374. 10.3201/eid0303.970317 9284385 PMC2627638

[B64] Sarantuya J. Nishi J. Wakimoto N. Erdene S. Nataro J. P. Sheikh J.. (2004). Typical enteroaggregative *Escherichia coli* is the most prevalent pathotype among *E. coli* strains causing diarrhea in Mongolian children. J. Clin. Microbiol. 42, 133–139. 10.1128/JCM.42.1.133-139.2004 14715743 PMC321701

[B65] Savarino S. J. Fasano A. Robertson D. C. Levine M. M. (1991). Enteroaggregative *Escherichia coli* elaborate a heat-stable enterotoxin demonstrable in an *in vitro* rabbit intestinal model. J. Clin. Invest. 87, 1450–1455. 10.1172/JCI115151 2010554 PMC295194

[B66] Savarino S. J. McVeigh A. Watson J. Cravioto A. Molina J. Echeverria P.. (1996). Enteroaggregative *Escherichia coli* heat-stable enterotoxin is not restricted to enteroaggregative *E. coli*. J. Infect. Dis. 173, 1019–1022. 10.1093/infdis/173.4.1019 8603943

[B67] Shamir E. R. Warthan M. Brown S. P. Nataro J. P. Guerrant R. L. Hoffman P. S. (2010). Nitazoxanide inhibits biofilm production and hemagglutination by enteroaggregative *Escherichia coli* strains by blocking assembly of AafA fimbriae. Antimicrob. Agents Chemother. 54, 1526–1533. 10.1128/AAC.01279-09 20086145 PMC2849362

[B68] Shin J. Oh S. S. Oh K. H. Park J. H. Jang E. J. Chung G. T.. (2015). An outbreak of foodborne illness caused by enteroaggregative *Escherichia coli* in a high school, South Korea. Jpn. J. Infect. Dis. 68, 514–519. 10.7883/yoken.JJID.2014.460 25971323

[B69] Steiner T. S. Lima A. A. Nataro J. P. Guerrant R. L. (1998). Enteroaggregative Escherichia coli produce intestinal inflammation and growth impairment and cause interleukin-8 release from intestinal epithelial cells. J. Infect. Dis. 177, 88–96. 10.1086/513809 9419174

[B70] Taddei C. R. Fasano A. Ferreira A. J. Trabulsi L. R. Martinez M. B. (2005). Secreted autotransporter toxin produced by a diffusely adhering *Escherichia coli* strain causes intestinal damage in animal model assays. FEMS Microbiol. Lett. 250, 263–269. 10.1016/j.femsle.2005.07.013 16098687

[B71] Vernacchio L. Vezina R. M. Mitchell A. A. Lesko S. M. Plaut A. G. Acheson D. W. (2006). Diarrhea in American infants and young children in the community setting. Pediatr. Infect. Dis. J. 25, 2–7. 10.1097/01.inf.0000195623.57945.87 16395094

[B72] Vial P. A. Robins-Browne R. Lior H. Prado V. Kaper J. B. Nataro J. P.. (1988). Characterization of enteroadherent-aggregative *Escherichia coli*, a putative agent of diarrheal disease. J. Infect. Dis. 158, 70–79. 10.1093/infdis/158.1.70 2899125

[B73] Vila J. Vargas M. Ruiz J. Espasa M. Pujol M. Corachán M.. (2001). Susceptibility patterns of enteroaggregative *Escherichia coli* associated with traveller's diarrhoea: emergence of quinolone resistance. J. Med. Microbiol. 50, 996–1000. 10.1099/0022-1317-50-11-996 11699598

